# Talking about chronic pain in family settings: a glimpse of older persons’ everyday realities

**DOI:** 10.1186/s12877-022-03058-8

**Published:** 2022-04-23

**Authors:** Gilles Merminod, Orest Weber, Imane Semlali, Anamaria Terrier, Isabelle Decosterd, Eve Rubli Truchard, Pascal Singy

**Affiliations:** 1 Department of Psychiatry, Liaison Psychiatry Service, Lausanne University Hospital, Lausanne, Switzerland; 2 Language and Information Sciences Department, Faculty of Arts, University of Lausanne, Lausanne, Switzerland; 3 Pain Center, Service of Anesthesiology, Lausanne University Hospital, Lausanne, Switzerland; 4 Faculty of Biology and Medicine, University of Lausanne, Lausanne, Switzerland; 5 Service of Geriatric Medicine and Geriatric Rehabilitation, Lausanne University Hospital, Lausanne, Switzerland; 6 Chair of Geriatric Palliative Care, Lausanne University Hospital, Lausanne, Switzerland

**Keywords:** Chronic pain, Older persons, Communication, Social network, Qualitative research

## Abstract

**Background:**

The expression of chronic pain remains a delicate matter for those older persons who suffer from this condition. If many studies highlight the difficulties of putting pain into words, scarce are those that take into account how given social networks can facilitate or prevent its expression. Based on a qualitative study that explores the communication about chronic pain in older persons’ social network, this article reports on this key issue of talking about health in later life within family settings and provides clinicians with information about the way older persons with chronic conditions perceive their everyday realities and social relations.

**Methods:**

A multidisciplinary research team (medicine, linguistics and psychology) interviewed 49 persons with chronic pain, all from the French-speaking part of Switzerland, aged 75 and older, without any major cognitive or auditory impairments. After transcription, the interviews were analyzed by combining content and discourse analysis with social network theories.

**Results:**

Communication about chronic pain depends significantly on the position of the interlocutors within the family structure, with a preference for direct relatives or individuals with similar difficulties. In social networks, the ability to communicate about chronic pain is both a resource (by allowing older persons to get help or by strengthening interpersonal relations) and a challenge (by threatening their autonomy, social relations or self-esteem).

**Conclusions:**

The study shows the predominance of the nuclear family (partner, children) in communication relating specifically to the everyday management of chronic pain. This state of affairs is, nevertheless, balanced by issues of (loss of) autonomy. These findings, in line with current trends in geriatrics, could benefit future reflections on the scope and limits of including relatives in the care of older patients with chronic conditions.

**Supplementary Information:**

The online version contains supplementary material available at 10.1186/s12877-022-03058-8.

## Background

Social relations are a crucial factor in healthy aging [1–3]. Families often play a key role in the wellbeing and continuous care of older family members. They provide help and emotional support [4], and can act as mediators between their older relatives and the healthcare structures [5]. This role is even more important in the case of those older family members with chronic health problems [6] who are not in permanent contact with the healthcare system. But, as a consequence, family members run the risk of being overwhelmed and exhausted [7].

The communication about chronic health issues within the family may face difficulties [8] even if family can play an important role in the decision-making relating to the treatment of chronic conditions, especially among older persons [9]. Difficulties in communication can lead to a reduction in a person’s ability to manage their health problems [10]. It is, therefore, critical to improve our understanding of the barriers and facilitators that weigh on older persons’ ability to communicate about chronic health issues with members of their social network. Too few studies have been systematically carried out on this issue, despite the fact that ageing is now a major demographic fact in many parts of the world [11].

Among the chronic health issues affecting older persons, chronic pain is one of the most common and has many consequences for their quality of life and social roles. It is, consequently, a case in point to study the facilitators and barriers to health communication in later life. If the expression of chronic pain is a difficult matter and requires specific language strategies at any age [12–15], older persons face many obstacles [16, 17]. General beliefs about the inevitability of pain in later life, stoicism and cautious attitudes may lead them to withhold information that could be useful for their treatment [18–22]. Age-related beliefs may also prevent the members of the older persons’ social network from taking into account what they say about chronic pain [23]. Chronic pain is an important factor of vulnerability among this demographic [24, 25] and impacts on the whole social environment of the persons who suffer from it [26, 27]. To date, despite an increased interest in the social environment of those older members of society who live with chronic health issues [22, 28], and the recognition of the need to integrate the resources of their social network into the care of chronic pain [29], research still provides scant information on the impact of an older person’s social environment on their ability to communicate about their pain [30].

Our study [31] examined the communication of chronic pain in older persons’ social network, which combines a multiplicity of actors such as family, friends, medical staff, etc. It aimed to give a glimpse of their everyday realities, to which health practitioners have little access, and also to have a better understanding of what it is like to live with chronic pain in later life. With this in mind, this article focuses on the communication of chronic pain in family settings. It is mainly driven by the following three questions: *(a)* With whom do older persons talk about their chronic pain in family settings? *(b)* What are the parameters that explain why some interlocutors are privileged over others? *(c)* What is it like to manage the topic of chronic pain within a family network?

## Methods

### Design & setting

This article reports parts of the results of a study on communication about chronic pain in the social networks of older persons. In this study, which was carried out by a multidisciplinary research team including medicine, linguistics, and psychology, we interviewed 49 persons over 75 years of age having chronic pain in order to examine their perceptions of the nature of communication about chronic pain and to identify their communicative needs. In order to do this, we adopted a qualitative approach that combined content and discourse analysis with qualitative social network analysis [31]. The study was carried out within the Lausanne University Hospital (Switzerland), and the research team was advised by a steering committee including medical and nursing staff, experienced researchers, and decision-makers working in the field of health in older populations.

### Data collection

We mainly recruited participants through members of the research team and the steering committee working with older persons, and through institutions such as day care centers, nursing homes, pain clinic, associations, and home care. For methodological reasons (feasibility and comparability of the interviews), the institutions and health practitioners working with us on the recruitment were instructed to check that the potential participants did not have major cognitive or auditory impairments. The recruitment of the participants was structured in three steps: (1) a person in contact with older people having chronic told them about the study and asked if the research team can contact them; (2) if so, a member of the research team called the potential participant, explained the ins and outs of the study, and asked if she wanted to participate; (3) if the person expressed interest, the member of the research team called her a week later to ask if she still wished to participle and, if so, to set a date for the first part of the interview. Our sample comprised 49 persons from the French-speaking part of Switzerland, aged 75 and older. They all suffer from chronic pain, here defined as pain that lasts more than three months [32]. We carried out the data collection by successive campaigns (from ten to ten).

The sample was built specifically to reflect the diversity of the older population in terms of sociodemographic variables (see Table 1), because social affiliations and trajectories affect health communication [31, 33]. We set a threshold of at least about 10 persons per group within a variable. It allowed us to reach saturation, with no new themes identified at the end of the research process.

**Table 1:** Respondents’ socio-demographic details

**Variables**		
*Gender*	Female	34
	Male	15
*Age*	75–85	28
	Over 85	21
*Primary socialization place*	Switzerland	34
	Other	15
*Socio-economic level*	Lower social class	31
	Upper social class	18
*Residence*	Home	40
	Nursing home	9
*Children*	Yes	39
	No	10
*Relationship status*	Single or widowed	33
	With partner	16

The research team carried out semi-structured interviews [34, 35] following an interview guide refined during the course of the study [36]. The interviews were divided into two parts, separated by a few days (at least 2 days, on average 7 days). The first part (45–60 min) documented the participants’ socio-biographic data (brief life history), a description of their chronic pain (type, duration, intensity, management, functional limitations, emotional and relational impact), and their social network. For this latter point, we used the concentric circle methods [37] that allowed the participants to map and order by degree of importance the members of their social network and to describe the nature of these social ties. The second part of the interview (45–60 min) examined the nature of communication about chronic pain with each member of the social network (frequency of interactions, contents and goals, difficulties and preferences, motivations and consequences, specific strategies to initiate or avoid the communication about chronic pain). It also investigated the specific needs of these older persons and expectations regarding communication and information about chronic pain.

### Data analysis

Following a grounded theory perspective [36], we have alternated successive interview campaigns with intermediate analyses. The interviews were transcribed and coded according to the principles of content analysis [38, 39]. The analysis was done with the qualitative data analysis software NVivo. Four researchers jointly carried out the coding while receiving a continuous feedback from the other members of the research team. The coding was inductive and relied on a process of intercoder agreement [31]. The analysis identified the semantic categories that are used by the participants to report and explain how they communicate about chronic pain within their social network. Discourse analysis supplemented content analysis: drawing on the tools developed in linguistics [40], the researchers were able to take into account participants’ ways of speaking (e.g., specific lexical choices, syntactic structures, discourse patterns) and to go beyond words’ literal meaning.

We coded the data so that the semantic categories correspond with the participants’ perspectives on social ties rather than a prior theorization. Drawing on the typical scenarios and role designators [41] used by our participants, we coded the data relating to three domains of social life that were mentioned predominantly by our participants when describing how they talk about chronic pain: the medical world (doctors, nurses, etc.), the family (partner, children, etc.) and their interactions with friends and acquaintances. We identified which domain was predominant by analyzing the social affiliation of the network’s members that were designated by the participants as playing an important or very important part in their life.

Within these three domains, we identified to whom the participants considered it appropriate to talk about chronic pain and for what reasons.

We distinguish three types of interlocutors: *main interlocutor*, *key interlocutor* and *potential interlocutor*. A *main interlocutor* is someone with whom the older persons say they talk (very) frequently about chronic pain. A *key interlocutor* is someone the older persons describe as being obviously needed when dealing with chronic pain. These two categories are not mutually exclusive: the same person may be both *main* and *key interlocutor*, and there may be a number of persons holding these communicative roles in the older person’s social network. Additionally, being a *main* or *key interlocutor* does not presuppose that the person is a good interlocutor. The category of *potential interlocutor* refers to a person with whom the older persons say they sometimes talk about chronic pain, without being either *main* or *key interlocutor*.

## Results

A strong trend in our data is that our respondents try to avoid communicating about their chronic pain with most of the members of their social network. Nevertheless, they generally talk about it with a selected set of interlocutors (see additional file 1). Most of them consider members of the medical profession (general practitioners and specialists, nurses, physiotherapists, etc.) to be *key interlocutors*, due to their expertise in health. *Key interlocutors* are often not the *main* ones. A large majority of the latter category are in the family, which is predominant in the social network of 38 participants. Being in a couple and having children is possibly related to having a predominantly family network: only one person with a partner and six persons with children did not have a predominantly familial social network. But it is not enough to be a member of the family network to be considered a *main interlocutor*. In addition to personal features, this position often goes hand in hand with a particular position in the family structure. *Main interlocutors* are generally among the direct relatives: partners and children. Siblings can function as *potential interlocutors*.

### Partners as default main interlocutors

In most cases, partners are identified as *main interlocutors* when it comes to talking about chronic pain. This is explained by their proximity and the reciprocal support that exists between partners who live together.

“The only person I can talk to is my wife [...] All we do is complain. She wakes up and says: ‘ouch! my feet’. Then I say: 'ow! today, my back'. But, anyway, it's a reflex.” (M76)

However, older persons may also hold back from communicating about chronic pain, either because they consider that their partner suffers more than they do and that it is therefore inappropriate to talk about their own problems, or because the partner is not particularly receptive. In the case of the latter, the communication about pain is seen as a threat to interpersonal relations.

“My husband, I hesitate to, I hesitate as much as possible to talk to him about my pain. Because it annoys him. Ah, it annoys him. Oh, talking to him about my pain is not allowed [...] Of course, sometimes, I complain to my husband, but he doesn't like that.” (W84)

There is also the fact that the partner may be affected by cognitive and physical troubles. Such problems can prevent the partner from being an appropriate interlocutor. For instance, when asked more precisely how the communication about pain with her husband is going, one of our participants remarked that she "…ask[s] him for help but… but one minute later it's forgotten." (W75) But the partner, even if absent, can remain an interlocutor or, at least, an audience to whom the participants can open up about their pain and the difficulties they encounter in daily life:

“I have my husband's ashes, which are there, in my room, on the night table. So I talk to him. I say: ‘Darling, you see where I stand. I'm all alone. I can't do it anymore’.” (W76)

It is not so much a response (of any kind) from the interlocutor that matters, but rather the possibilities for communication (complaining, confiding, etc.) that this form of interpersonal relationship allows.

### Children as frequent main interlocutors

Children are the family members most mentioned by our participants, even before partners. This is probably due to the fact that three quarters of the participants in our research have children, whereas only a little over a third have a (still living) partner. Children usually have the role of *main interlocutors*. They are often the most important persons to talk to, even though several of our respondents insist on the fact they do not want their health problems to weigh on their children. This aspect is well illustrated by one of our participants who reports on the reaction of her daughter after she told her about her health problems:

“My daughter […] she’s immediately so sorry! It makes me sad because she already has enough problems.” (W75)

The frequency of contact the children have with their older parents facilitates the communication about chronic pain, as the following extract shows:

“When it hurts, it hurts. I can't do otherwise. [...] I don’t display it. My daughter tells me: ‘I can hear in your voice that you are not well’ […] She tells me: ‘Mum, you are not well’. So, sometimes, I tell her. I don't always want to worry her. She tells me: “No, Mum, you're not well, you mustn't tell me that you're well, because I can hear it in your voice’.” (W76)

This familiarity with the parent’s usual way of being – developed through repeated, often daily, contact over a long period of time – gives children the ability to pick up on cues about their parent’s health without the latter having to communicate it explicitly. The key role given to these cues is that they allow the issue of chronic pain to be addressed without the older person having to initiate it. Activities such as changing medication and introducing new care procedures can also be opportunities to address the issue of pain, either through the request for, or the offer of, advice.

Among our participants daughters are more likely to act as the *main interlocutors* than sons. This does not prevent the latter from fulfilling this role, particularly when they have comparable health problems. In-laws – while generally considered to be fully part of the family – are not designated as the m*ain interlocutors*, with the exception of a few cases of particularly caring daughters-in-law.

The parents’ level of autonomy has an impact on the way they communicate about chronic pain with their children. Two cases can be distinguished schematically in our data. The first case is that of the autonomous person.

« I don't talk about it, even to my son, even to those who are closer to me. I don't talk about everything, just because I don't want to worry them […] Usually, when it hurts a lot, I don't talk about it. I take a painkiller, and it gets better. But I don't say anything because I'm too scared to worry them. [...] If I had pain in a way I never had before, then I would ask a doctor before telling my son […] I prefer to warn him when I know that something is serious. I prefer to warn him when I am sure.” (W100)

In this case, the older person displays all the attributes usually associated with autonomy, including the ability to express one’s own beliefs and values and to make decisions freely. Concerning communication, this means that the person has room in terms of what he or she wishes to reveal to their relatives and how much leeway is given to them. It is, therefore, largely up to the parent to decide whether or not to make their children an interlocutor in the communication about chronic pain.

The second case is that where the older person is dependent on (one of) their children.

« My daughter […] helps me with all the paperwork, and my medication, and everything. For example, yesterday, I was not feeling well at all. So she contacted me: ‘If there is anything, Mum, I'll come straight away’ [...] I was in so much pain. [...] Then she told me: ‘Mum, we've been to the pain management clinic’. And they had given me cannabis drops, and I had stopped taking cannabis because it's very expensive [...] So she told me: ‘If you feel that you don't need it any more, you stop, you should stop it’. So I stopped. And yesterday, I was in so much pain, she told me: ‘What have you done with your drops, Mum?’ I said: ‘I've kept them’. ‘Then take three drops!’ I took three drops last night and this morning.” (W88)

In such a case, children often act as caregivers, providing relational, logistical and medical assistance to their parents, who are losing a part of their autonomy. In this context, children, as caregivers, appear to be legitimized to incite or even enjoin their parents to carry out particular actions and not only to support or advise them. They are not just *main interlocutors*, but they can act as if they have the ability to act upon what their parents do.

Distant relatives (nephews, nieces, cousins, etc.) are not generally mentioned by our participants as persons to whom they speak about chronic pain. Nevertheless, in some cases, distant relatives – often younger than the older person such as nephews or nieces – can take on the role of caregivers usually assigned to the partner or children in our data. They are generally presented as *main interlocutors* in the communication about chronic pain.

“If I have a crisis, if my niece is upstairs, I call her, she comes. [...] The person who is closest to me on these things is my niece who lives upstairs, because she likes medicine so much.” (W83)

These persons fill a sort of structural role left vacant by the absence of a child or partner. Nevertheless, in our data, administrative or financial dependency on a distant relative seems to inhibit communication about chronic pain.

In most cases, our participants say that they do not talk about the pain they are experiencing with their grandchildren. They express the will to share positive experiences with them or to help them but not to show them their own difficulties. It is, therefore, generally left to the children (i.e., the parents of the grandchildren), as *main interlocutors* and, thus, mediators, to inform the grandchildren about the health of their grandparents.

### Siblings as potential interlocutors

In our data, our respondents do not actively seek to talk about chronic pain with their siblings, who are, nevertheless, generally considered as potential interlocutors. In some cases, siblings are seen as particularly appropriate interlocutors because they share a similar life experience. This creates a feeling of belonging to the same community, namely those who suffer from chronic pain in later life.

“My sister often comes to visit me here. She has taken care of me a lot, so we can exchange a lot. She lost her husband, and now she comes more often. So, it's an exchange (...) We exchange on our pain, our views (...) We encourage each other.” (W90)

Similarly, another participant emphasized that she can talk about her pain with her brothers, “…because both of them are also disabled” (W80). Communication, in these cases, seems to lead to emotional support.

It should also be mentioned that some distant relatives appear to be potential interlocutors, particularly when they are of a similar age, such as cousins but also nephews or nieces in big families. For instance, a participant, who said that she did not communicate about chronic pain with distant relatives (“I'm not going to dump my pain on my nephews and nieces”), made an exception for nieces of the same age with whom she was raised:

“My niece A* […] she's not well […] I can talk about it [chronic pain] with her, yes, on the phone. That's also how I talk to these two nieces, B* and C*. [...] With this niece, C*, with her, we can talk. Really. Deeply. Really.” (W79)

As is the case with siblings, there is a sense of belonging here based on a common life history and fed by similar experiences of chronic pain. In the case of reduced mobility, however, which frequently affects older persons having chronic pain, it is sometimes difficult to find a means other than the telephone to communicate with members of their social network.

## Discussion

Despite recurring difficulties, the participants talk about chronic pain with a diversity of interlocutors within their social network: health professionals, family and friends. According to our data, family is usually favored by our participants. Furthermore, within the family, direct relatives (partners and children) are often preferred over other interlocutors. The context of caregiving and care-receiving relationship may qualify this preference. It raises issues of autonomy, which become particularly delicate when the relationships with others (notably direct relatives) are redefined by health difficulties and sometimes reversed (as in the case of adult children caring for their parents).

In our study, the difficulties associated with the expression of chronic pain mainly relate to the risk of threatening social relations and damaging self-esteem. Most of our participants said that they wanted to avoid raising this topic within their social network. By doing so, they can exist as social actors beyond the daily experience of chronic pain, and prevent their social interactions from revolving around pain and illness exclusively, as observed in other contexts [42, 43]. The fact of not talking about chronic pain more generally reflects a dynamic of accommodation on the part of older persons, maximizing their social desirability [44] and avoiding their stigmatization [45]. For instance, not talking about it with grandchildren appears symptomatic of a desire to avoid falling into stereotypes of intergenerational communication in which older persons are often depicted as verbose and focused on their aging problems [46].

The participants in our study tend to select the interlocutors that are worth speaking to, particularly those who can respond to biomedical, psychological or social needs. This selection seems to reflect a careful management of the available resources. As research has repeatedly shown [47–51], the expression of chronic pain often has a negative impact on family members, especially if they do not know what information to retain or how to react to it. Nevertheless, even if chronic pain often has a deleterious effect on familial relationships [52], talking about it with specific family members can be an opportunity to strengthen interpersonal relations [53] and to find social support to live with chronic pain in everyday life [54]. The selection of specific interlocutors leads to a division of labor in the social network [55]: the communication about chronic pain with direct relatives is usually motivated both by their ability to provide practical help and to be emotionally supportive. Such a division of labor seems to be based on the traditional model of family in Western societies, although family models have now changed considerably [56, 57]. The individual’s structural position in the social network does not simply and unequivocally determine the way the older person communicates about chronic pain. As shown in our data, shared affinities and experiences play a central role. Social networks are multiplex realities [58] echoing the multilayered nature of identities in communication [59].

The study of the communication about chronic pain within older persons’ social networks also sheds light on the reconfiguration of autonomy in later life [60–62], especially relating to family. In bioethics, autonomy is the patient’s right to self-determination and free choice [63]. From a functional perspective, autonomy is the individual’s ability to carry out their daily activities independently [64]. Since chronic pain is often associated with functional decline [65], those older persons with chronic pain negotiate parts of their autonomy in a transforming environment, where a relationship as equals runs the risk of being reconstituted as a dependent relationship. Autonomy is thus nested in the relational ecology that forms the older person’s social network [66, 67]. In this regard, for older members of the population, the very fact of choosing their interlocutors can be a way of exercising their autonomy. In a life made up of constraints and affected by the loss of physical or cognitive abilities, the choice of whether or not to talk about their pain can be understood as a space in which they still have some leeway. In some cases, dependence on a family member, especially in the management of medication, may lead to more frequent communication about pain, but this dependence – or the risk of becoming dependent – may also inhibit communication by threatening the older person’s identity of having control over their fate [68].

In summary, this study has given a glimpse of the everyday realities experienced by older persons having chronic pain, relaying their voices and drawing attention to their own concerns. This is all the more important given that older persons may be silenced or may themselves consider that they no longer have any say in the matter. Behind the communication about chronic pain, there are more general issues about how family and social networks can be a resource for dealing with health problems in the everyday realm. Such a situation comes with its challenges for older persons: having access to such a resource also means knowing how to manage it. That being said, our study has limitations that should be addressed by further research. For example, it would benefit from a comparable study focusing on the relatives of older persons with chronic pain, because communication is always a joint action which involves at least two parties. In addition, further research should integrate persons with dementia or cognitive troubles as this would necessarily weigh on communication [69]. Furthermore, an ethnographic study documenting older persons’ actual practices of communication in their daily lives would probably lead us to reconsider our results and would show that a significant part of the communication relating to chronic pain is based on non-verbal resources [70].

## Conclusion

The analysis of the different types of interlocutors within the family illuminates two significant dimensions that are both opportunities and challenges for communication about chronic pain in later life: on the one hand, the need to protect social ties despite pervasive, continuous health problems; on the other hand, the need for autonomy, especially when the individuals are in a position where they may become more and more dependent on family members. Being aware of these issues can help health professionals open up the discussion with older persons on the benefits, or not as the case may be, of talking about chronic pain in family settings, knowing that it can be a resource just as much as a challenge. We now hope that similar studies will be carried out in other parts of the world, providing data from cultures that are even more, or far less, in line, with traditional family models.

## Supplementary Information

**Additional file 1. **Main extracted themes.

## Data Availability

The datasets generated and analyzed during the current study are not publicly available to preserve the anonymity of participants but are available from the corresponding author on reasonable request.
