# Supplementary material for: Talking about chronic pain in family settings: a glimpse of older persons’ everyday realities
Source: BMC Geriatr. 2022 Apr 23;22:358. doi: 10.1186/s12877-022-03058-8 (PMC9034600; doi:10.1186/s12877-022-03058-8)
Supplement: Supplementary file 1 — Additional file 1. Main extracted themes. [file 12877_2022_3058_MOESM1_ESM.docx]

**Additional file 1 : main extracted themes**

|  |
| --- |
| 1. **Interlocutors with whom older persons talk about chronic pain**    1. **Health practitioners**   doctor  nurses  nurses’ aides  social workers  members of home health care service  physiotherapist  caregivers  podiatrist  pharmacist  psychiatrist  masseur   - 1. **Family members**   children  wife  husband  family  daughter-in-law  sister  brother  cousin  nieces and nephews  grandchildren   - 1. **Friends and acquaintances**   friends  members of the fibromyalgia association  residents of the nursing home  members of the neighborhood association  neighbors |
| 1. **Themes discussed with the interlocutors** |
| cause of pain |
| intensity of pain |
| location of pain |
| feelings related to pain |
| pain of others |
| impact of pain |
| pain management |
| 1. **Facilitators of communication about chronic pain** |
| - 1. **Older persons’ self-motivations** |
| being in pain |
| needing information |
| being talkative |
| - 1. **Interlocutors’ features facilitating communication** |
| having the same experience of chronic pain |
| being of the same age |
| being of same gender |
| having expertise |
| being an intimate |
| - 1. **Interlocutors’ behaviors facilitating communication** |
| showing interest |
| showing empathy |
| showing willingness to listen |
| 1. **Barriers to communication about chronic pain** |
| - 1. **Older persons’ self-restraints** |
| considering chronic pain as age-related (banalization) |
| considering chronic pain as a private matter |
| having been educated not to talk about chronic pain (stoicism) |
| being worried of being boring |
| being worried of being annoying |
| being worried of worrying others |
| - 1. **Interlocutors’ features inhibiting communication** |
| not having any experience of chronic pain |
| being younger |
| being of a different gender |
| having cognitive impairments |
| having auditory impairments |
| lacking of health literacy |
| - 1. **Interlocutors’ behaviors inhibiting communication** |
| showing no empathy |
| showing no willingness to listen |
| showing no communication abilities |
